# H1N1 2009 pandemic flu vaccination campaign: The Homeless lesson

**DOI:** 10.1371/currents.RRN1146

**Published:** 2010-02-03

**Authors:** Philippe Brouqui, Jean-Christophe Lagier, Nadim Cassir, Sékéné Badiaga, Hans Gadelius

**Affiliations:** ^*^Service des Maladies Infectieuses et Tropicales, CHU Nord AP-HM , URMITE CNRS/IRD 6236, faculté de médecine, Université de la méditérranée, Marseille; ^†^Service des Maladies Infectieuses et Tropicales, Assistance Publique Hopitaux de Marseille, Hôpital Nord, Marseille, France, URMITE, CNRS 6236 and ^‡^AP-HM, Marseille, France

## Abstract

Homeless are deprived people of developed countries that have a particularly low vaccine coverage and are exposed to vaccine preventable infectious diseases. We report here the efficiency of a voluntary based one-day snapshot influenza vaccination in homeless shelter of Marseille, France, which allowed to obtain a 46.9% H1N1 pandemic vaccine coverage while at the same time only 6% of the French population has been vaccinated.

Homeless are deprived people of developed country that are particularly exposed to infectious diseases [1] . They are usually non medical seeker and vaccine preventable contagious disease outbreaks such as hepatitis A , diphtheria, tuberculosis and influenza have already been reported this population [1] . (H1N1) 2009 pandemic flu is ongoing at time we write and in France H1N1 (2009) vaccine coverage is low and approximately 4 million peoples (6%) have receive the vaccine yet. Within French health care workers, after a 4 weeks campaign, vaccine coverage is about 37% with 68% medical doctors and only 20% in nurses (*personal unpublished data*). In such situation we wonder what would be the knowledge on pandemic flu, the acceptability of vaccination and the H1N1 (2009) vaccine coverage obtainable in the homeless population we work with since 1999 [2] . 

            We already reported the effectiveness of using a mobile chart strategy to improve the seasonal flu vaccine coverage in health care workers and we demonstrated that snapshot intervention is a very efficient strategy to improve health care in the homeless population [3] [4] . Consequently we decided to propose a voluntary based one-day snapshot influenza vaccination in homeless shelter of Marseille France. 

On, December 12^th^ 2009 a team of 5 infectious diseases doctors, 3 interns and 2 students went to visit à 300 bed capacity homeless shelter carrying Pandemrix® (GSK) adjuvanted vaccine, and  Mutagrip® (Sanofi Pasteur MSD) 2009 seasonal flu vaccine. People living in the shelters were informed three days earlier of the vaccination campaign. Volunteers only were included. All participants have read approved and signed a consent form. As there is a public health value in doing so, WHO recommended seasonal influenza and pandemic influenza vaccines can be administered together [5] . Consequently, once consent was obtained, people were questioned and vaccinated with either Pandemrix® or Mutagrip® or both. A specific questionnaire was filled up and captured in the French data base as recommended by the French Ministry of Health to insure traceability and a vaccination certificate was given back to the people once vaccinated.

Pandemrix® vaccine was given to 117/249 (46.9%) homeless people that reach the shelter this given night, 26 of them being vaccinated against both seasonal and pandemic flu. One homeless was not vaccinated because of contraindication (anticoagulant therapy), two were proposed a second shoot at 3 weeks because there were immunocompromised.   Male homeless represented 92.3%. Among the 118 homeless 61.4% declared to have had tetanus vaccine, 51% diphtheria and poliomyelitis, 24% hepatitis. Interestingly among volunteers almost all (96.2%) were aware of the benefit of influenza preventive vaccination and none questioned on possible side effect. Recently mandatory vaccination by using tracker system has been proven efficient in obtaining 88% pandemic H1N1 (2009) vaccine coverage [6] . Here we demonstrate that mobile chart and snapshot intervention are an efficient strategy to obtain good vaccine coverage in the homeless.

            In conclusion, while French Pandemic vaccination coverage is of 6% in the general population, homeless living in a Marseilles shelter achieved a vaccine coverage of 46.9%. While generally non medical seeker, homeless are more afraid against the disease than against possible vaccine side effect. This is a lesson that we need to consider.  

 **Funding information**The authors thank the Conseil Général 13, for providing for free the Mutagrip® seasonal influenza Vaccine, the pandemrix® being provided free of charge by the French Ministry of Health.

**Competing interests**The authors have declared that no competing interests exist.

**Authors' contributions**PB wrote the text, SB organized the snapshot, JCL , NC and HG have token part in the vaccination campaign.
